# Long-term methotrexate efficacy in juvenile localized scleroderma

**DOI:** 10.1186/1546-0096-9-S1-O16

**Published:** 2011-09-14

**Authors:** F Zulian, C Vallongo, F Vittadello, G Zanon, S Giuliotto, G Martini

**Affiliations:** 1 Rheumatology Unit, Department of Pediatrics, University of Padua, Padua, Italy

## Background

Recent studies reported that methotrexate (MTX), appears beneficial in juvenile localized scleroderma (JLS) but little is known about its long-term efficacy. We assessed the long-term efficacy of MTX in a cohort of patients with JLS.

## Methods

We prospectively followed a cohort of patients with JLS who were enrolled in a double-blind, randomized controlled trial. Oral MTX was used at a dose of 15 mg/m^2^ once a week (max 20 mg) for at lest 24 months; prednisone (1 mg/Kg/day, max 50 mg), in a single morning dose for 3 months was added. A target lesion was evaluated clinically, with infrared thermography and using a computerized scoring system with skin score rate (SSR) evaluation. Response to treatment was defined as: no new lesions; SSR<1; decrease lesion temperature by at least 10% compared to baseline. Treatment failure was defined by new lesions, SSR>1, or increased lesion temperature. Partial Remission (PR) was defined when the state of responder was maintained ON treatment for at least 6 months, **c**omplete remission (CR) the state of responder OFF treatment for at least 6 months.

## Results

65 patients have been treated with MTX during the open-label phase of the study. Seven patients were lost to follow-up. Of the remaining 58 patients, after a mean follow-up of 43 months (median 36; range 6-72 mesi), 48 (82.8%) were responders, 10 (17.2%) relapsed by 24 months since MTX start. Among the responders, 35 (60.4%) after a MTX treatment for 27.5 months (median 24, range 18-30) maintained CR for 25 months (median 24, range 2-48). None of those in CR relapsed. 13 patients (22.4%), after a mean follow-up of 20.5 months (median 15.5, range 6-45), were in PR.

## Conclusion

Methotrexate shows a prolonged efficacy in patients with JLS.

